# Temperature-Controlled Direct Imprinting of Ag Ionic Ink: Flexible Metal Grid Transparent Conductors with Enhanced Electromechanical Durability

**DOI:** 10.1038/s41598-017-11475-8

**Published:** 2017-09-11

**Authors:** Yong Suk Oh, Hyesun Choi, Jaeho Lee, Hyunwoo Lee, Dong Yun Choi, Sung-Uk Lee, Kyeong-Soo Yun, Seunghyup Yoo, Taek-Soo Kim, Inkyu Park, Hyung Jin Sung

**Affiliations:** 1 Department of Mechanical Engineering, Korea Advanced Institute of Science and Technology, Daejeon, 34141 Korea; 2 Department of Electrical Engineering, Korea Advanced Institute of Science and Technology, Daejeon, 34141 Korea; 3 Powder & Ceramics Division, Korea Institute of Materials and Science, Changwon, 51508 Korea

## Abstract

Next-generation transparent conductors (TCs) require excellent electromechanical durability under mechanical deformations as well as high electrical conductivity and transparency. Here we introduce a method for the fabrication of highly conductive, low-porosity, flexible metal grid TCs *via* temperature-controlled direct imprinting (TCDI) of Ag ionic ink. The TCDI technique based on two-step heating is capable of not only stably capturing the Ag ionic ink, but also reducing the porosity of thermally decomposed Ag nanoparticle structures by eliminating large amounts of organic complexes. The porosity reduction of metal grid TCs on a glass substrate leads to a significant decrease of the sheet resistance from 21.5 to 5.5 Ω sq^−1^ with an optical transmittance of 91% at λ = 550 nm. The low-porosity metal grid TCs are effectively embedded to uniform, thin and transparent polymer films with negligible resistance changes from the glass substrate having strong interfacial fracture energy (~8.2 J m^−2^). Finally, as the porosity decreases, the flexible metal grid TCs show a significantly enhanced electromechanical durability under bending stresses. Organic light‐emitting diodes based on the flexible metal grid TCs as anode electrodes are demonstrated.

## Introduction

Transparent conductors (TCs) are indispensable in a variety of optoelectronic devices, including organic light-emitting diodes (OLEDs), organic solar cells, and touch screen panels^1^. Indium tin oxide (ITO)-based films have been the industrial standard for a long time due to their low electrical sheet resistance (*R*_s_) and high optical transmittance (*T*); however, ITO-based films include several drawbacks, including inherent brittleness and the need for expensive sputtering processes^2^. Alternative materials to ITO-based films, including conducting polymers^3^, graphene^4^, carbon nanotubes^5^, random metal nanowire networks^6–10^, and regular metal grids^11^, have been reported for use in flexible optoelectronic devices. Among these materials, regular metal grids offer many advantages, such as facile control over their grid width and spacing, scalability to large-area application and low junction resistance^12, 13^.

Cost-effective and solution-processed metal grid TCs on flexible substrates have been reported using two different fabrication schemes: (1) grid-patterned cavity is formed into a polymer substrate using hot embossing process and then is filled with metal nanoparticle (NP) ink^14–16^. This method facilitates the fabrication of metal grid structures with the relatively high aspect ratio (=height/width), while they should be sintered at low temperature (<150 °C) due to thermal degradation of the polymer substrate; (2) metal NP structures on a glass substrate are fabricated using a variety of wet deposition methods, such as direct imprinting (DI)^17, 18^, gravure printing^19^, electrohydrodynamic jet printing^20, 21^ and inkjet printing^22^. Although this method is not suitable for fabricating the high aspect ratio structures of metal NPs, the metal grid TCs on the glass substrate can be sintered at high-temperatures (>200 °C) to improve the electrical conductivity^23, 24^. After high-temperature sintering process, the metal grid TCs are transferred from the glass substrate to the polymer substrate for the fabrication of flexible metal grid TCs.

Among the wet deposition methods, the DI of colloidal metal NPs can directly produce micro/nanoscale metal structures at low-costs and in a high-throughput manner without expensive etching steps and metal evaporation^25, 26^. Recently, our group suggested a reservoir-assisted DI method based on an Ag ionic ink, which has several advantages such as higher metal content and less aggregation than colloidal metal NPs, for the fabrication of high-performance flexible metal grid TCs^17, 18^. However, the Ag NP structures fabricated by the thermal decomposition of Ag ionic ink include numerous micro/nanoscale pores due to the elimination of organic complexes^27, 28^. Unfortunately, those pores can deteriorate electrical and mechanical properties of the flexible metal NP structures^29–33^. First, the formation of many pores precludes the generation of highly conductive metal grid TCs. Second, since high-temperature sintering process (~300 °C) induces strong interfacial fracture energy (IFE) between the metal and the substrate, micro/nanoscale pores can cause serious damage to metal NP structures during the transfer process. Finally, as the cracks are initiated and propagated near numerous pores under mechanical deformations, the flexible metal grid TCs may be easily damaged by static or dynamic bending stresses. The formation of micro/nanoscale pores inside the metal NP structures should be minimized in the development of flexible optoelectronic applications.

In order to resolve this problem, we introduce a novel temperature-controlled direct imprinting (TCDI) process of Ag ionic ink based on two-step heating for the generation of highly conductive, low-porosity, flexible metal grid TCs. This TCDI technique led to stably capturing the Ag ionic ink and reducing the porosity of thermally decomposed Ag NP structures. The electrical resistivity (*ρ*_m_) and *R*_s_ of the metal grid TCs at a fixed transmittance (*T*_550nm_) at 550 nm were represented as a function of the organic complex contents and geometrical calculation. The effect of porosity on the transfer process of metal grid TCs to the polymer film was explored at different sintering temperatures. In addition, the effect of porosity on an electromechanical durability of the flexible metal grid TCs was examined under the static and dynamic bending stresses. Finally, the utility of flexible metal grid TCs as anode electrodes was demonstrated by fabricating of OLEDs.

## Results

### Temperature-controlled direct imprinting of Ag ionic ink

**Figure 1:**
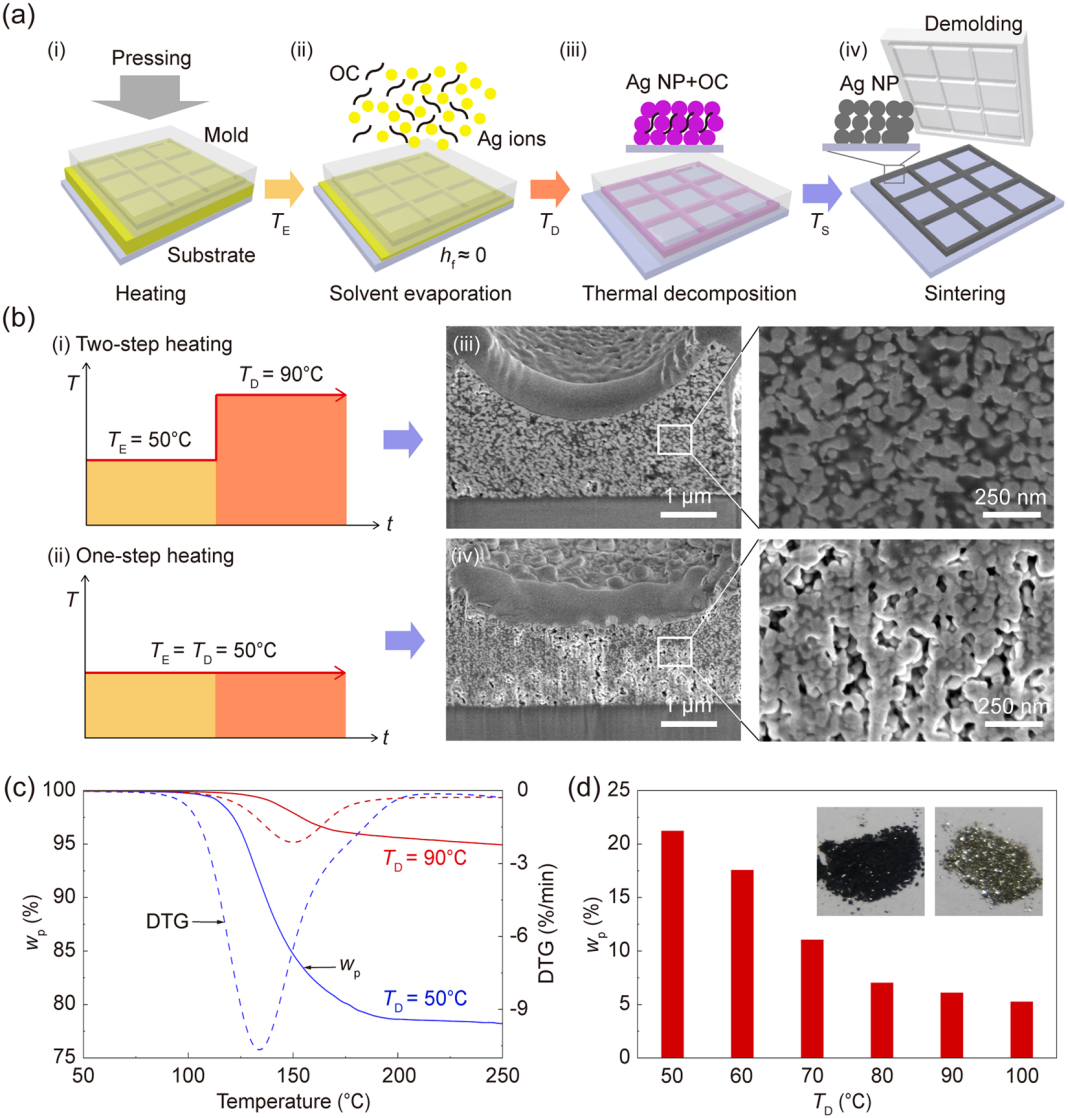
(**a**) The TCDI of Ag ionic ink. (**b**) (i-ii) Schematic illustration of the two-step heating of TCDI and the one-step heating of typical DI, respectively. (iii-iv) Cross-sectional FIB-SEM images and magnified SEM images of the metal grid line structures fabricated using TCDI and DI of Ag ionic ink, respectively. (**c**) TGA and DTG curves obtained from the metal-organic complex powders as a function of *T*_D_. (**d**) Weight percent of metal-organic complex powders, as a function of *T*_D_. The inset shows photographs of the metal-organic complex powders, dried at two different values of *T*_D_ = 50 and 90 °C for 3 hour, respectively.

The TCDI of Ag ionic ink, which involves the two-step heating, is schematically illustrated in Fig. 1a: (i) Ag ionic ink (10 μL) was imprinted on a fluorinated glass substrate using a grid-patterned mold under low pressure (*P* = 120 kPa) and low temperature; (ii) the first heating step was performed at an evaporation temperature (*T*_E_) of 50 °C over 5 min to stably capture the Ag ionic ink inside the grid-patterned cavity. It should be noted that when the Ag ionic ink is initially heated at an increased *T*_E_, large amounts of the filled ink leave from the grid-patterned cavity through a liquid film between the mold and the substrate due to carbon dioxide generation during the thermal decomposition of Ag ions^34^; (iii) for a liquid film with negligible thickness (*h*_f_ ≈ 0), the second heating step was carried out at decomposition temperature (*T*_D_) = 90 °C to eliminate organic complexes derived from the thermal decomposition of the Ag ions and to improve their thermal decomposition rate; (iv) after complete solvent evaporation, the grid-patterned mold was carefully removed from the substrate. The Ag NP structures were thermally treated at sintering temperature (*T*_S_) = 300 °C for 10 min. Figure 1b shows a schematic illustration of (i) the two-step heating of TCDI and (ii) the one-step heating of typical DI, respectively. In Fig. 1b(iii-iv), focused ion beam-scanning electron microscope (FIB-SEM) images show a cross-section of the metal grid line structures fabricated using TCDI and DI of Ag ionic ink, respectively. As *T*_D_ was increased from 50 °C to 90 °C, the micro/nanoscale pores in the metal grids were significantly reduced. This micro/nanoscale pores may be mainly generated by the elimination of organic complexes during the thermal decomposition process. Amounts of organic complexes inside the Ag NP structures were compared using thermogravimetric analysis (TGA) and derivative thermogravimetric (DTG) analysis of Ag NP-organic complex powders, respectively. Figure 1c shows the TGA and DTG curves obtained from Ag NP-organic complex powders after complete drying at *T*_D_ = 50 and 90 °C, respectively. The Ag NP-organic complex powders dried at the lower *T*_D_ showed the larger weight loss and the faster decomposition rate in the temperature range of 100–200 °C. The Ag NP-organic complex powders dried at *T*_D_ = 50 and 90 °C showed a weight loss of 21.24 and 6.17%, respectively. These results indicated that the metal grids generated using the DI of Ag ionic ink may include a greater fraction of the organic complex by weight, about 15% more, than those obtained using the TCDI of Ag ionic ink. Figure 1d plots a weight loss of the Ag NP-organic complex powders dried at different values of *T*_D_. As *T*_D_ increased from 50 to 100 °C, the organic complex contents of the powder decreased. The inset shows photographic images of the metal-organic complex powders. The Ag NP-complex powders dried at *T*_D_ = 50 °C showed dark black due to high organic complex contents (left), while those obtained at *T*_D_ = 90 °C showed bright silver due to relatively low organic complex contents (right).

### Highly conductive, low-porosity metal grid TCs

**Figure 2:**
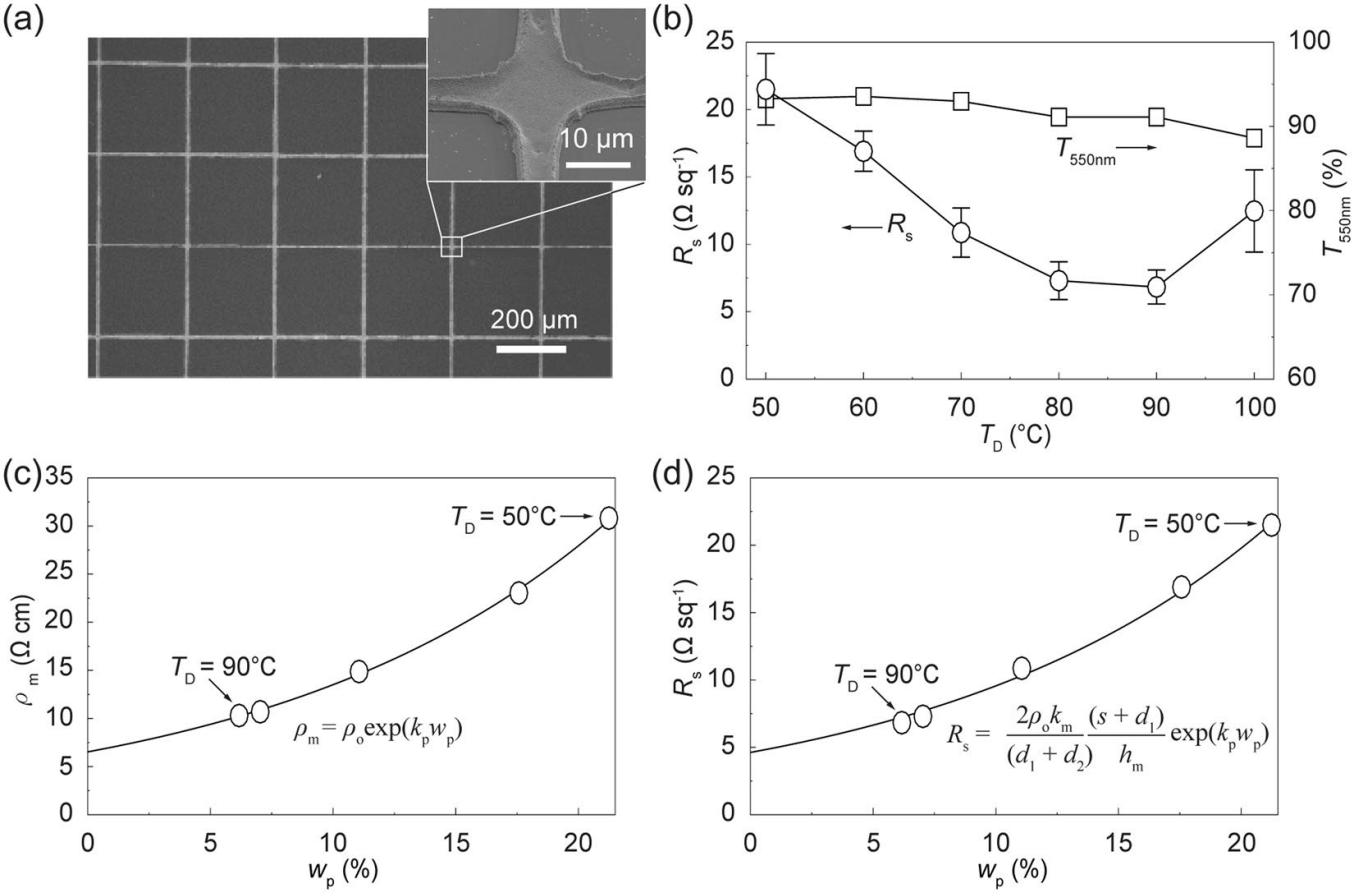
(**a**) A SEM image of the metal grid TCs fabricated using the TCDI of Ag ionic ink. The inset shows a magnified SEM image of the intersection in the Ag NP structure. (**b**) Sheet resistance of the metal grid TCs generated at different values of *T*_D_. (**c**) Electrical resistivity of the Ag NP structures as a function of the organic complex weight. (**d**) Sheet resistance of the metal grid TCs as a function of the organic complex weight.

Figure 2a shows a SEM image of the Ag NP structures fabricated over the grid-patterned mold area (10.5 mm × 10.5 mm). The presence of unwanted residual layers within the grid spacing was negligible. The inset shows a magnified SEM image of the Ag NP structures at the intersection of metal grids. A linewidth (~8 μm) of the metal grid was smaller than that of the original grid-patterned cavity (15 μm) due to mold deformation. Figure 2b shows the values of *R*_s_ and *T*_550nm_ for the metal grid TCs generated using the TCDI of Ag ionic ink. As *T*_D_ increased from 50 to 90 °C, *R*_s_ of the metal grid TCs decreased significantly, holding *T*_550nm_ over 91.2%; however, the value of *R*_s_ for the metal grid TCs fabricated at *T*_D_ = 100 °C increased and fluctuated as solvent evaporation and thermal decomposition occurred near the boiling point of the base-solvent (toluene, 110.6 °C). Figure S1(a) shows transmittance spectra over a wavelength range of 350–800 nm of the metal grid TCs fabricated at different values of *T*_D_. The spectral transmittance of metal grid TCs fabricated at *T*_D_ = 100 °C did significantly decrease from 350 to 500 nm. Also, figure of merit (FoM), which generally predicts the performances of TCs, was compared for the metal grid TCs generated at different values of *T*_D_ in Figure S1(b). As *T*_D_ increased to 90 °C, the FoM of the metal grid TCs was increased from 250 to 572. The value of *ρ*_m_ of thermally reduced Ag NP line structures fabricated at different values of *T*_D_ is compared in Fig. 2c. The value of *ρ*_m_ could be expressed using the following relationship:

1 $${{\rho }}_{m}({x})={\rho }_{o}\exp ({{k}}_{{\rm{p}}}{{w}}_{{\rm{p}}}),$$

where *ρ*_o_ is the electrical resistivity of the thermally reduced metal structures without organic complex contents, *w*_p_ is the weight percent of organic complex and *k*_p_ is a correction factor from experimental data. The correction factor was determined to be *k*_p_ = 0.0727. The value of *ρ*_o_ (6.5287 × 10^−6^ Ω cm) was 4 times larger than that of the bulk Ag (1.59 × 10^−6^ Ω cm).

The *R*_s_ of the metal grid TCs was calculated based on the structural size and organic complex weight. The equation used to predict *R*_s_ was constructed by modifying the expression for *R*_s_ obtained using a square wire network, as suggested by Van de Groep *et al*.^13^. The cross-section of the Ag NP structures was regarded as trapezoidal, and *R*_s_ was predicted using the following equation:

2 $${{R}}_{s}=\frac{2{{\rho }}_{m}{{k}}_{m}}{({{d}}_{{\rm{1}}}+{{d}}_{{\rm{2}}})}\frac{({{s}}_{m}+{{d}}_{{\rm{1}}})}{{{h}}_{m}}=\frac{2{\rho }_{o}{{k}}_{m}}{({{d}}_{{\rm{1}}}+{{d}}_{{\rm{2}}})}\frac{({{s}}_{m}+{{d}}_{{\rm{1}}})}{{{h}}_{m}}\exp ({{k}}_{{\rm{p}}}{{w}}_{{\rm{p}}}),$$

where *h*_m_ is the thickness of the metal grid line, *d*_1_ is the bottom width and *d*_2_ is the top width of the metal grid line, and *s* is the spacing between the metal grid lines. The value of *k*_m_ depends on a difference between the predicted shape of the Ag NP structures and their actual shape. The mean values of *d*_1_, *d*_2_, *h*_m_, *s*_m_ for the metal grid line were 8 μm, 6 μm, 2 μm and 257 μm, respectively. The correction factor, extracted from experimental results, was determined to be *k*_m_ = 3.74. Figure 2d indicates that the *R*_s_ of the metal grid TCs fabricated at different values of *T*_D_ could be suitably fit to the theoretical values.

### Transfer process of metal grid TCs

**Figure 3:**
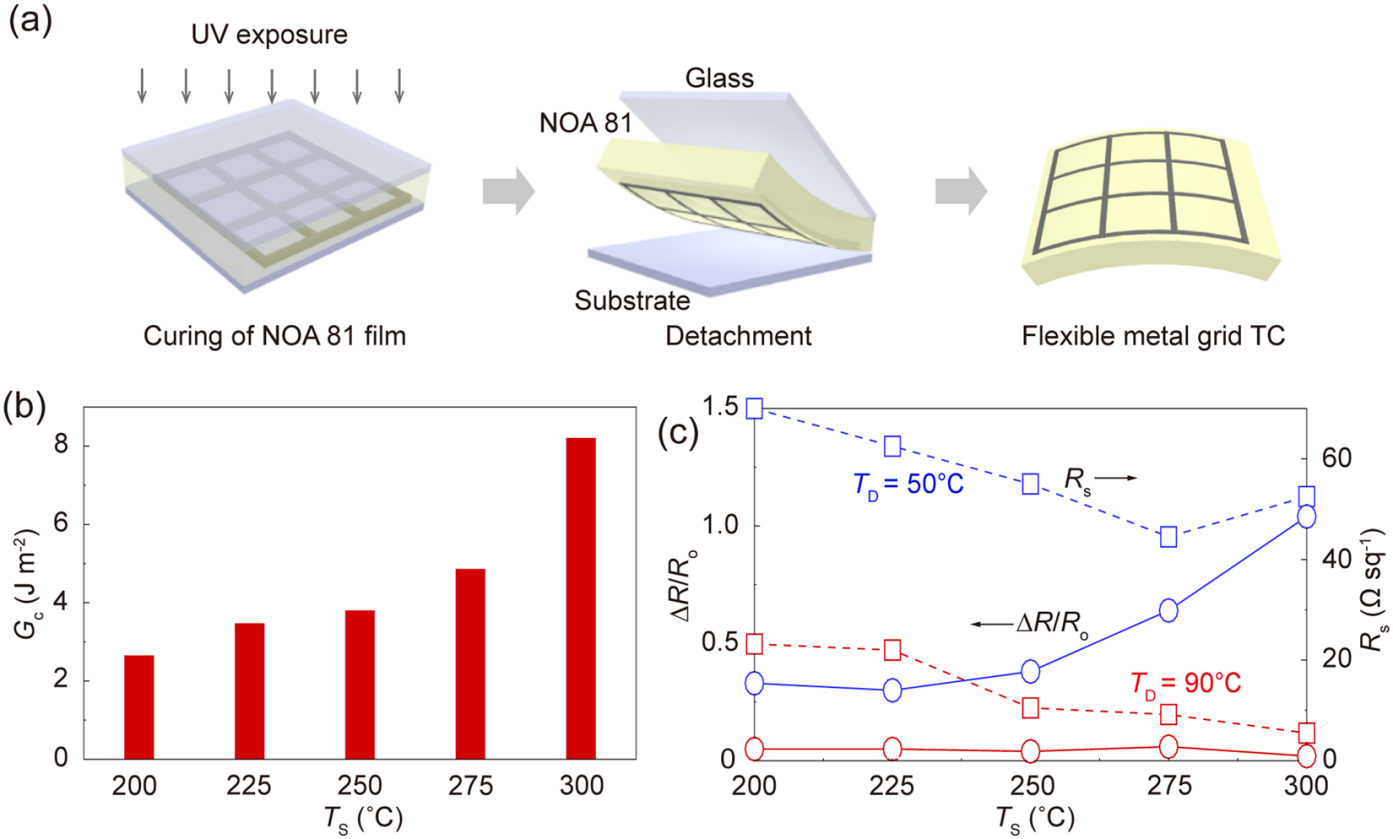
(**a**) Schematic illustration of the process used to transfer the metal grid TC from the glass substrate to the transparent polymer film (NOA 81 film). (**b**) Interfacial fracture energy of the Ag NP film sintered at different values of *T*_S_. (**c**) Normalized resistance changes of the metal grid TCs and the flexible metal grid TCs after the transfer process. The resistance of the flexible metal grid TCs sintered at different *T*_S_ for comparison.

The metal grid TCs on the glass substrate should be transferred onto the polymer substrate for use in flexible optoelectronic devices. A transfer process of metal grid TCs provided the flexibility and significantly reduced the surface roughness that can cause optoelectronic device failure. Figure 3a shows a schematic illustration of the transfer process, based on a sandwich structure (glass substrate/polymer film/glass substrate) to generate a uniform, thin and transparent polymer film on both sides. It should be noted that when the polymer film becomes thinner, the flexible metal grid TCs show better electromechanical durability under the same bending radius (*r*) due to a decrease of the nominal bending strain^35^. The Norland Optical Adhesive 81 (NOA 81), which shows a high optical transparency over a wide spectral range with a good mechanical flexibility and a strong adhesion force to metal structures, is used as the polymer film. The NOA 81 solution was poured onto the metal grids-patterned glass substrate, and then was covered by the glass substrate. After the UV exposure, the metal grids embedded within NOA 81 film were detached from two glass substrates.

The effect of the porosity reduction on the transfer process of metal grid TCs was explored by measuring the resistance changes of metal grid TCs sintered at different values of *T*_S_. The higher *T*_S_ induces the more increase of the IFE between the metal and the glass substrate, which causes serious damage to the flexible metal grid TCs during the transfer process. Especially, the metal structures with many pores are prone to be broken due to the increase of the IFE. In Figure S2(a), the IFE of Ag NP film on the glass substrate was quantitatively measured using a double cantilever beam (DCB) fracture mechanics testing method at different values of *T*_S_. In Figure S2(b), the inset shows a schematic diagram of the DCB test specimen consisting of a fluorinated glass substrate (donor substrate), a Ag NP film, an adhesion layer, and the plasma-treated glass substrate (receiving substrate). Figure 3b plots the IFE between the Ag NP film and the substrate after *T*_S_ over the range 200–300 °C. Although the glass substrate was treated by the fluorine, the IFE between the Ag NP film and the glass substrate depended strongly on *T*_S_. The Ag NP films sintered at *T*_S_ = 300 °C, in particular, displayed 3-fold larger IFE (~8.2 J m^−2^) than those sintered at *T*_S_ = 200 °C (~2.7 J m^−2^). Figure 3c shows the normalized resistance change (∆*R*/*R*_o_) and *R*_s_ of the flexible metal grid TCs after the transfer process of the metal grid TCs sintered *T*_S_ = 200–300 °C. As *T*_S_ increased, ∆*R*/*R*_o_ of the flexible metal grid TCs based on the DI of Ag ionic ink (*T*_D_ = 50 °C) significantly increased from 33% to ~104% due to an increase of the IFE. On the other hand, the flexible metal grid TCs obtained using the TCDI of Ag ionic ink (*T*_D_ = 90 °C) displayed a small value of ∆*R*/*R*_o_ (~6%). Although the IFE increased, the porosity reduction led to preventing a significant increase in ∆*R*/*R*_o_ from serious mechanical damage. The *R*_s_ of the flexible metal grid TCs fabricated at *T*_D_ = 50 and 90 °C showed 5.5 and 52.5 Ω sq^−1^, respectively.

### Highly conductive, low-porosity, flexible metal grid TCs

**Figure 4:**
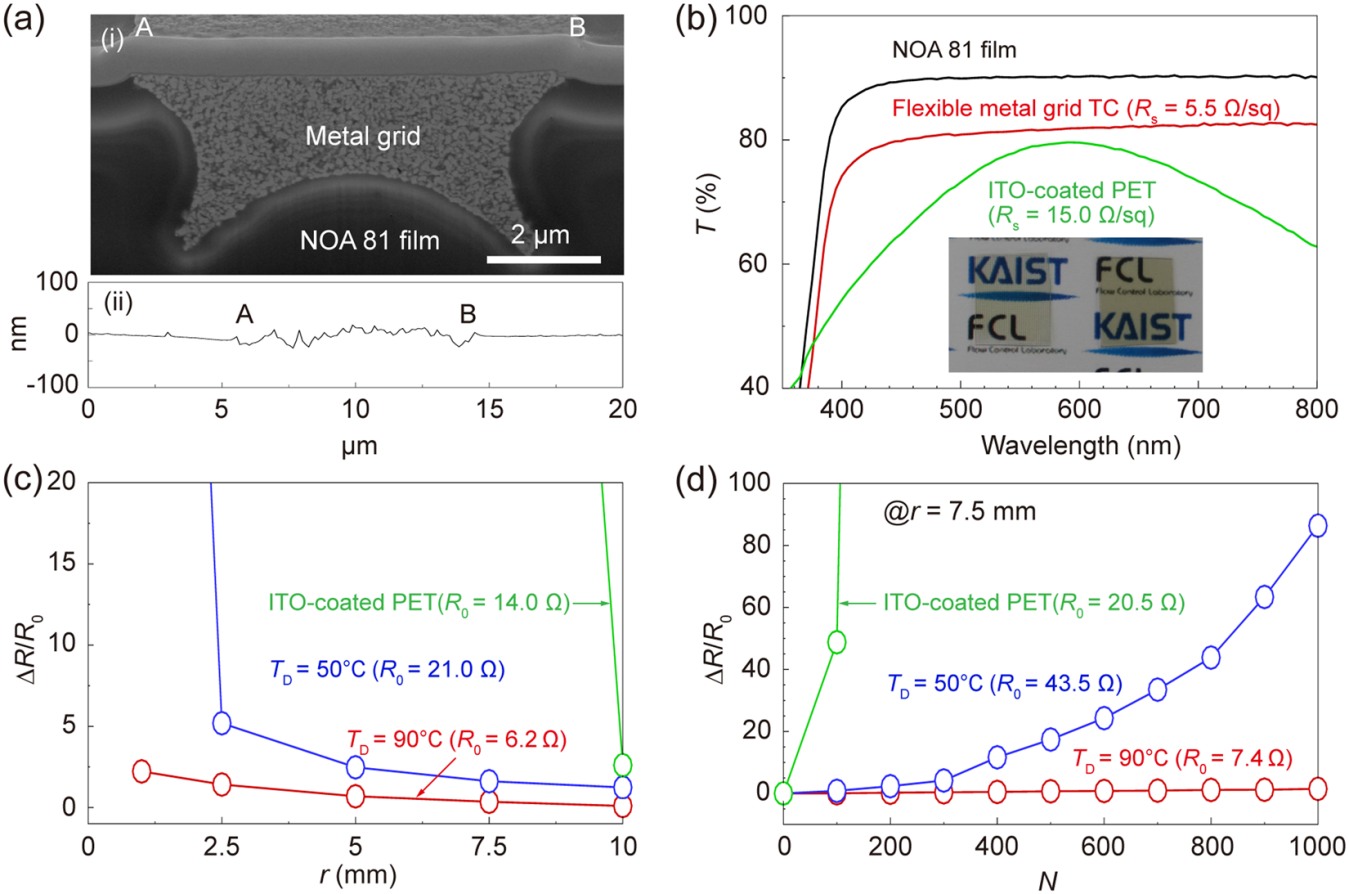
(**a**-**i**) A cross-sectional surface of the metal grid TCs-embedded within the NOA 81 film. (**a**-**ii**) AFM surface profile of the metal grids TCs within the NOA 81 film. (**b**) Transmittance spectra over a wavelength range of 350–800 nm, of the NOA 81 film, the metal grid TCs-embedded within the NOA 81 film, and the ITO-coated PET film, respectively. The inset shows photographs of the metal grids TC-embedded NOA 81 film (left) and ITO-coated PET film (right). (**c**) Normalized resistance changes of the flexible metal grid TCs (*T*_D_ = 50 and 90 °C, respectively) and ITO-coated PET film as a function of the bending radius. (**d**) Normalized resistance change of flexible metal grid TCs (*T*_D_ = 50 and 90 °C, respectively) and ITO-coated PET film during the dynamic bending tests.

Figure 4a(i) shows a cross-sectional FIB-SEM image of the metal grid-embedded the NOA 81 film. The metal grid structures were buried below the NOA 81 surface. Figure 4a(ii) shows an atomic force microscopy (AFM) surface profile of the flexible metal grid TCs, which presented smooth surfaces with a root-mean-square surface roughness of 6.7 nm and a maximum peak-to-valley value of 43.8 nm. Figure 4b compares the values of *T* and *R*_s_ for the flexible metal grid TCs with the values obtained from a commercially available ITO-coated polyethylene terephthalate (PET) film. Here, it should be noted that the transmittance spectra of the bare NOA 81 film, the flexible metal grid TCs, and the ITO-coated PET film include the transmittance through the substrate. The flexible metal grid TCs performed better (*R*_s_ of 5.5 Ω sq^−1^ and *T*_550nm_ of 81.47%) than the ITO-coated PET film (*R*_s_ of 15.0 Ω sq^−1^ and *T*_550nm_ of 78.35%). The *T* of the ITO-coated PET film fluctuated over the wavelength range of 350–800 nm, whereas the corresponding values of the flexible metal grid TCs remained constant.

The electromechanical durability of the flexible metal grid TCs under static and dynamic bending stresses is an important factor to be considered in flexible optoelectronic devices^35, 36^. As the number of pores in the metal grid structures decreased, the mechanical durability of the flexible metal grid TCs can be improved by inhibiting pore-induced crack initiation and propagation under bending stresses^29–33^. This resulted in reducing the electrical resistance changes of the metal grid structures under bending stresses. Figure 4c plots the values of ∆*R*/*R*_o_ obtained from the flexible metal grid TCs fabricated using TCDI and DI of Ag ionic ink under a static bending test. The value of ∆*R*/*R*_o_ of the ITO-coated PET film under a static bending test was evaluated as a reference. As the flexible metal grid TCs were bent up to *r* = 1 mm, the values of Δ*R*/*R*_o_ increased for these two flexible metal grid TCs (*R*_o_ = 6.3 and 21.0 Ω sq^−1^) by a factor of 2.2 and 113.3 respectively. The *R*/*R*_o_ of the flexible metal grid TCs fabricated using the DI of Ag ionic ink significantly increased at *r* = 1 mm due to serious damage. Figure 4d plots the values of ∆*R*/*R*_o_ obtained from the flexible metal grid TCs fabricated using TCDI and DI of Ag ionic ink under a dynamic bending test with *r* = 7.5 mm. The value of ∆*R*/*R*_o_ of the ITO-coated PET film under a dynamic bending test was evaluated as a reference. After the only several bending cycles, the resistance of the ITO-coated PET film significantly increased to a few tens of kΩ due to formation of numerous and visible cracks. After 1000 cycles of repeated bending/relaxation, ∆*R*/*R*_o_ obtained from the flexible metal grid TCs (*R*_o_ = 7.4 and 43.5 Ω sq^−1^) increased by a factor of 1.5 and 86.4, respectively. When *T*_D_ increased, the porosity reduction of the flexible metal grids led to decreasing ∆*R*/*R*_o_ under static and dynamic bending stresses (a significant decrease in the relative ratio of resistance change by more than 51 times at *r* = 1 mm and by more than 57 times after the 1000 bending cycles, respectively). The low-porosity, flexible metal grid TCs fabricated using the TCDI of Ag ionic ink showed an excellent electromechanical durability under static and dynamic bending tests, indicating their great potential for use in various flexible optoelectronic devices.

## Discussion

### Applications to flexible organic light-emitting diodes

**Figure 5:**
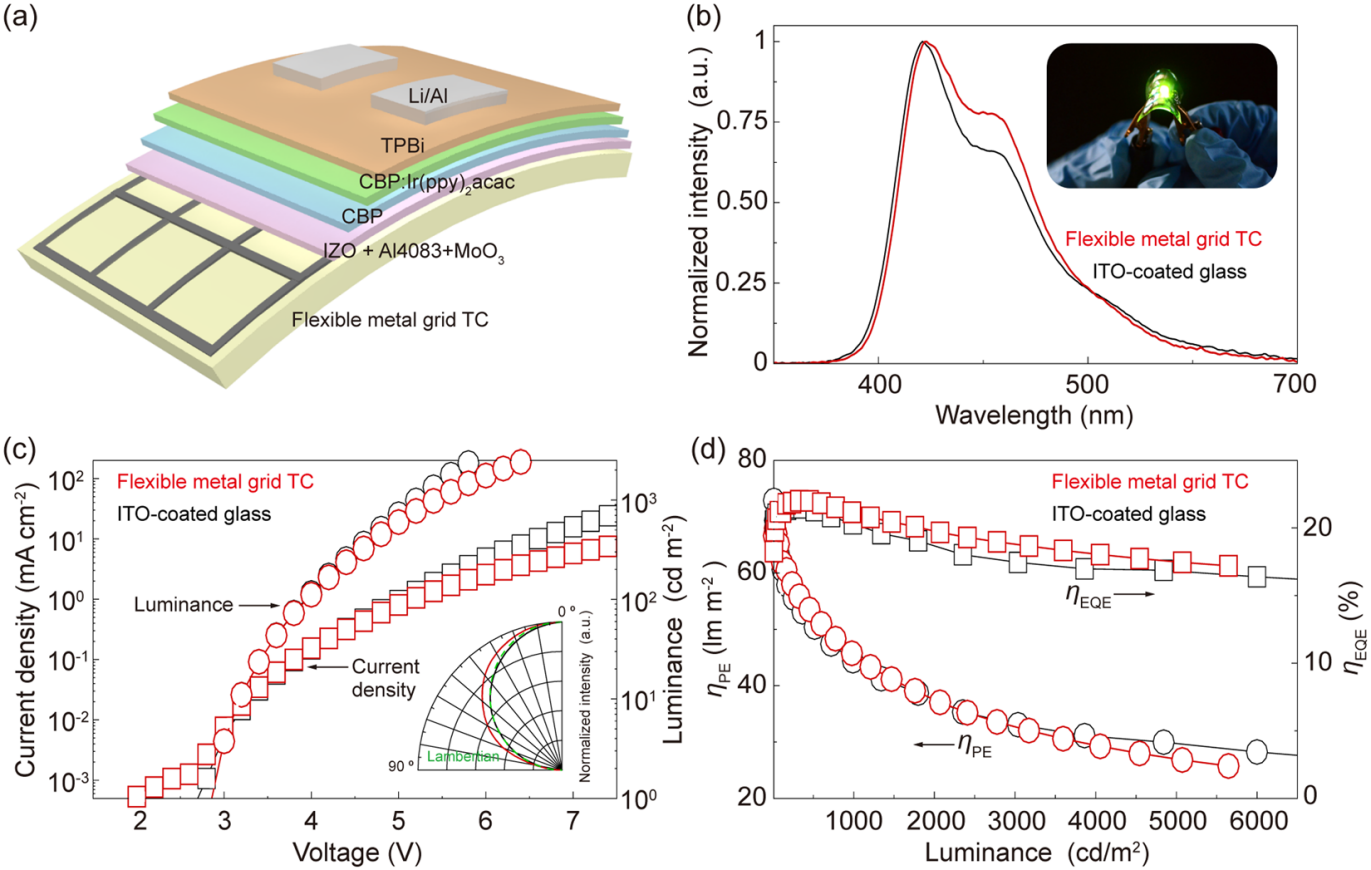
(**a**) Schematic illustration of the flexible OLEDs prepared using the embedded metal grid TC. (**b**) The normalized intensity spectra of the flexible OLEDs. The inset shows a photograph of the flexible OLEDs working at high luminance. (**c**) Current density−voltage characteristics and luminance-voltage characteristics of the proposed flexible OLEDs and reference cells on ITO-coated glass substrates. The inset shows the angular dependences of the normalized intensity. (**d**) Power efficiency and external quantum efficiency vs luminance.

Figure 5a shows a schematic diagram of OLEDs based on the flexible metal grid TCs. Indium zinc oxide (IZO) layers with a thickness of 50 nm were used to cover the flexible metal grid TCs, which exhibit *R*_s_ of 5.5 Ω sq^−1^ and *T*_550nm_ of 81.47%. Poly(3,4-ethylenedioxythiophene) polystyrene sulfonate (PEDOT:PSS) and MoO_3_ layers were used on the top of IZO films for efficient hole injection. The 4,4′-Bis(N- carbazolyl)-1,1′-biphenyl (CBP) layer doped with green phosphorescent emitters of bis(2-(2-pyridinyl-N)phenyl-C)(acetylacetonate) iridium (III) (Ir(ppy)_2_acac, 7 wt%) was used as an emitting layer. The CBP and 2,2′,2″-(1,3,5-Benzinetriyl)-tris(1-phenyl-1-H-benzimidazole) (TPBi) layers were used as hole- and electron- transport layers, respectively. Figure 5b shows the normalized electro-luminescence spectrum of the flexible OLEDs in the normal direction in the wavelength range of 350–700 nm, which is similar to that of the ITO-based TCs. The inset shows the operating image of the flexible OLEDs under bending. The OLEDs fabricated on the flexible metal grid TCs show an electrically stable operation due to their relatively good uniformity and low roughness, as shown in Fig. 5c. The inset shows the angular emission profile, which was used to obtain the external quantum efficiency (*η*_EQE_) and power efficiency (*η*_PE_) of the devices. Therefore, the OLEDs based on the flexible metal grid TCs show the comparable values of *η*_EQE_ and *η*_PE_ with those of ITO-based OLEDs, as shown in Fig. 5d. The flexible OLEDs exhibit *η*_EQE_ of 22.0% and *η*_PE_ of 61.8 lm W^−1^ at 111.6 cd m^−2^, while ITO-based OLEDs show *η*_EQE_ of 21.5% and *η*_PE_ of 60.6 lm W^−1^ at 116.2 cd m^−2^, respectively.

In summary, we demonstrated the fabrication of highly conductive, low-porosity, flexible metal grid TCs *via* the TCDI of Ag ionic ink. As *T*_D_ increased during the thermal decomposition of Ag ionic ink, the porosity of metal grid structures was significantly reduced due to the elimination of large amounts of organic complexes. Also, both *ρ*_m_ and *R*_s_ of metal grid TCs were estimated based on the weight percent of organic complex and geometrical calculations. The porosity reduction led to improving the optoelectrical properties of metal grid TCs (*R*_s_ < 6 Ω sq^−1^ at *T*_550nm_ = 91%) and preventing serious damage (∆*R*/*R*_o_ < 6%) from the strong IFE between the metal and the substrate during the transfer process. In addition, we verified that the porosity reduction resulted in significantly enhancing an electromechanical durability of the flexible metal grid TCs under static and dynamic bending stresses. The OLED based on flexible metal grid TCs as anode electrodes was demonstrated. The uniformity, reliability and scalability of the metal grid TCs should be further improved by the combination of uniform pressing-stable detaching system and the exact control of fluidic properties (viscosity and surface tension) before this technology can be commercialized. We believe that this strategy can provide a useful approach to enhance optoelectrical properties and an electromechanical durability of solution-processed metallic TCs for the development of next-generation flexible optoelectronic devices.

## Methods

### Ag ionic ink

The Ag ionic ink (TEC-IJ-060, Inktec) consisted of Ag ions (Ag alkyl carbamate complexes), a base solvent (methanol and toluene) and additives. Prior to the TCDI of Ag ionic ink, methanol was evaporated at 100 °C for 10 min to improve the concentrated ink filling. The Ag alkyl carbamate complexes were decomposed to Ag NPs, carbon dioxide, and the corresponding alkyl amines by heating above 50 °C for a few minutes^34^.

### PDMS mold

The PDMS solution (Sylgard 184, Dow Corning) was generated by mixing the silicon elastomer kit and a curing agent (10:1), and this mixture was poured onto a SU-8 master. After PDMS curing at 100 °C for 1 hour, a PDMS mold was carefully released from the SU-8 master. In the PDMS mold, the grid-patterned cavity was designed with a width (*w*) of 15 μm and a spacing (*s*) of 250 μm by calculating the geometrical shadow zone, *T* = *s* ^2^/(*s* + *w*)^2^. The value of a cavity height was limited to 7.5 μm to prevent the destruction of Ag NP structures during the detachment of PDMS mold. However, the value of *T*_550nm_ measured from the metal grid TCs was higher than that obtained using the geometrical calculation of grid-patterned mold due to mold deformation (93.2 and 89%, respectively).

### Effect of pressure

The effect of *P*, ranged from 30 to 300 kPa, was examined by considering the Poiseuille’s law (*h*_f_^4^ is inversely proportional to ∆*P*)^37^. At 30 kPa, most of the ink was not captured inside the grid-patterned mold due to a large value of *h*_f_. The ink was effectively captured inside the mold cavity over 100 kPa. The pressure was optimized at 120 kPa by comparing *Rs* and *T*_550nm_ of the metal grid TCs. Also, the relatively uniform *P* based on horizontal levels led to decreasing both *R*_s_ and resistance fluctuations of the metal grids.

### Transfer process

All types of glass substrate, including soldalime glass and borosilicate glass, were treated using 1 H, 1 H, 2 H, 2H-perfluorooctyl-trichlorosilane (448931, Sigma-Aldrich) for 3 min in a vacuum chamber. After the sintering process, a metal grid-patterned glass substrate (soldalime) was re-treated with the fluorinated silane. The NOA 81 solution was poured onto the metal grid-patterned glass substrate, and it was covered by a borosilicate glass substrate. After UV exposure, the borosilicate glass substrate was detached from the NOA film, and then the metal grid-embedded within NOA 81 film (its thickness of 150 μm) was detached from the sodalime glass substrate.

### Fabrication of flexible organic light-emitting diodes

The IZO film (~50 nm) was deposited on the flexible metal grid TCs using an RF sputtering system (RF power: 120 W). The PEDOT:PSS (Clevios PVP AI4083, Heraeus, 45 nm) layers were spin-coated on the top of the IZO films. PEDOT:PSS was coated also on pre-coated ITO glass substrates (<12 Ω sq^−1^, AMG Inc., Korea) as a reference after O_2_ plasma treatment (70 W, 1 min). The samples with PEDOT:PSS were dried to remove any residual solvent at 100 °C for 10 min on the hot plate. Finally, the PEDOT:PSS-coated samples were loaded into a thermal evaporator for deposition of organic, metal oxide layers, and metal electrodes under high vacuum (3 × 10^−6^ Torr) conditions. The stacked multilayers of OLEDs were composed of MoO_3_ (10 nm)/CBP (20 nm)/CBP doped with 7 wt% of Ir(ppy)_2_acac (20 nm)/TPBi (55 nm)/LiF (1 nm)/Al (100 nm).

### DCB test

The Ag NP film/glass substrate (sodalime) specimens were fabricated with the size of 37.5 mm × 9 mm. The specimens were sandwiched by an additional glass beam using an epoxy (353ND, Epoxy Technology). The final structure of the DCB test specimen was the glass substrate/epoxy/Ag/glass substrate. The connection parts with the DCB test machine and aluminum loading tabs were attached to the specimens with the selective epoxy (DP420, 3M) and the specimens were cured at 120 °C for 1 h in a convection oven.

### Characterization

The images of the metal grid TCs were measured using field-emission SEM (S-4800, Hitachi). Cross-sectional images of the metal grid TCs were measured using FIB-SEM (Helios Nanolab 600, FEI), and the surface roughness of the flexible metal grid TCs was measured using AFM (XE-100, Park Systems). The transmittance spectra were measured using a UV-VIS-NIR spectrophotometer (Lambda 1050, Perkin-Elmer). The *R*_s_ of the metal grid TCs was measured using the two-terminal method and four-point probe method (4200-SCS, Keithley)^38^. Two electrodes between the metal grids, separated by a square area (25 mm^2^), were fabricated using conductive pens (CW2200MTP and CW2900, ITW Chemtronics).

## Electronic supplementary material

Supplementary Information
