# Supplementary material for: Temperature-Controlled Direct Imprinting of Ag Ionic Ink: Flexible Metal Grid Transparent Conductors with Enhanced Electromechanical Durability
Source: Sci Rep. 2017 Sep 11;7:11220. doi: 10.1038/s41598-017-11475-8 (PMC5593849; doi:10.1038/s41598-017-11475-8)
Supplement: Supplementary file 1 — Supplementary Information [file 41598_2017_11475_MOESM1_ESM.doc]

Supplementary Information

Temperature-Controlled Direct Imprinting of Ag Ionic Ink: Flexible Metal Grid Transparent Conductors with Enhanced Electromechanical Durability

Yong Suk Oh,^1^ Hye Sun Choi,^1^ Jaeho Lee,^2^ Hyunwoo Lee,^2^ Dong Yun Choi,^3^ Sung-Uk Lee,^1^ Kyeong-Soo Yun,^1^ Seunghyup Yoo,^2^ Taek-Soo Kim,^1^ Inkyu Park,*^1^ & Hyung Jin Sung*^1^

*^1^Department of Mechanical Engineering, Korea Advanced Institute of Science and Technology, Daejeon 34141, Korea, E-mail:* [*hjsung@kaist.ac.kr*](mailto:hjsung@kaist.ac.kr) *E-mail:* [*inkyu@kaist.ac.kr*](mailto:inkyu@kaist.ac.kr)

*^2^Department of Electrical Engineering, Korea Advanced Institute of Science and Technology, Daejeon 34141, Korea*

*^3^Powder & Ceramics Division, Korea Institute of Materials and Science, Changwon 51508, Korea*


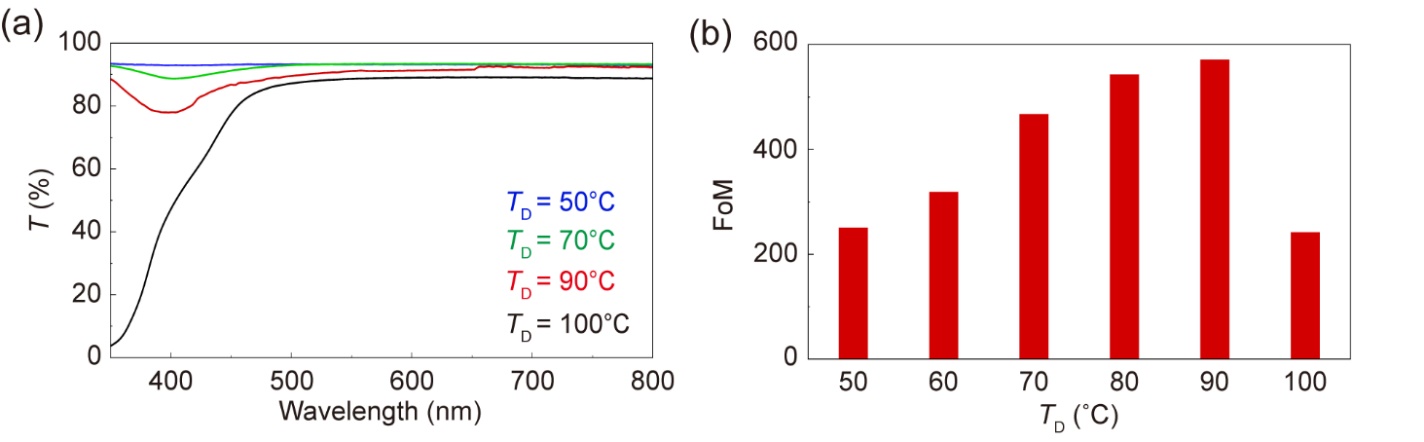


Figure S1. (a) Transmittance spectra over a wavelength range of 350–800 nm of the metal grid TCs fabricated using TCDI of Ag ionic ink at different values of *T*_D_. (b) Comparison of FoM values for the metal grid TCs.

Figure S1(a) shows transmittance spectra over a wavelength range of 350–800 nm of the metal grid transparent conductors (TCs). As *T*_D_ increased from 50 to 90°C, the spectral intensity did slightly decrease near a wavelength range of 350-500 nm. However, the metal grid TCs fabricated at *T*_D_ = 100°C showed a significant reduction of the spectral intensity near a wavelength of 400 nm due to unwanted residual layers within the grid spacing.

The optoelectrical properties of TC-like thin film could be described using the following equation:[^1^](#_ENREF_1)

$T=\left(1+\frac{188.5}{R_{\text{s}}}\frac{\sigma_{\text{opt}}}{\sigma_{\text{dc}}}\right)^{^{-2}},$ (1)

where σ_dc_ is the direct current conductivity and σ_opt_ is the optical conductivity of the film. In Equation (1), the value of σ_dc_/σ_opt_ is a figure of merit (FoM) that predicts the performance of the TCs. In Figure S1(b), the FoM of the metal grid TCs fabricated using the TCDI of Ag ionic ink (*T*_D_ = 90°C) is 572, which is significantly higher than those obtained using flexible TCs based on the alternative materials, including conductive polymer (<60),[^2^](#_ENREF_2) carbon nanotube (<90),[^3^](#_ENREF_3) graphene (<120)[^4^](#_ENREF_4) and Ag nanowire (<160).[^5^](#_ENREF_5)


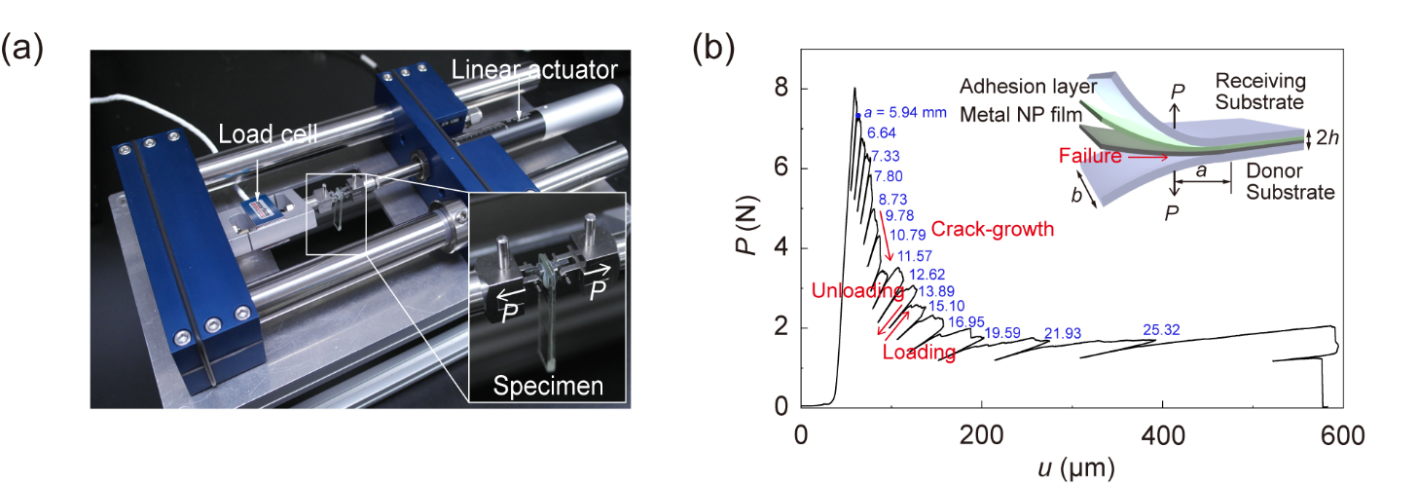


Figure S2. (a) DCB fracture mechanics testing system consisting of a load cell and a linear actuator. (b) Load-displacement curve for the DCB test.

In general, the adhesion energy can be measured using a variety of methods, including the peel test,[^6^](#_ENREF_6) die shear test,[^7^](#_ENREF_7) four point bending test,[^8^](#_ENREF_8) and double cantilever beam (DCB) test.[^9-11^](#_ENREF_9) Among others, the DCB test is suitable for measuring the adhesion energy of various thin films, such as a nanoporous organosilicate thin film and a graphene layer. Lee *et al*.[^9^](#_ENREF_9) showed that the interfacial fracture energy (IFE) of a metal NP film could be increased by forming the organic residual bridges between the metal NPs, depending on the sintering temperature and time. The adhesion energy between the metal NPs and the substrate was examined by measuring the IFE, using the DCB fracture mechanics testing method. Figure S2(a) shows that the DCB fracture mechanics testing system (Delaminator Adhesion Test System, DTS Company) consisted of a linear actuator and a load cell. A glass substrate is generally used as an ideal elastic bending beam for DCB tests.[^10^](#_ENREF_10) The thin film sandwiched between two glass substrates was initially loaded elastically. Once the strain energy exceeded the IFE between the Ag NP film/fluorinated glass substrate, a crack-growth occurred at the film-glass interface. At this critical point, the slope of the load displacement curve decreased, reflecting a change in the specimen compliance (*C*) as a function of the crack extension. In Figure S2(b), the inset shows a schematic diagram of the DCB test specimen. Figure S2(b) plots the debond lengths (*a*) calculated over multiple loading/crack-growth/unloading cycles during the DCB test, using the slope of the cycle (d*u*/d*P*), that is, the value of *C* in Equation (2):[^12^](#_ENREF_12)

$a=\left(\frac{CE'bh^{3}}{8}\right)^{1/3}- 0.64h.$ (2)

Here, *u* is the total displacement of the beam ends, *P* is the applied load, *E’* is the plane strain modulus of the beam (*E’* = 76 GPa), *b* is the sample width (*b* = 9 mm), and *h* is the half height of the substrate (*h* = 1 mm). The critical load (*P*_c_) is the load at which the load-displacement curve deviates from linearity during the crack-growth within the loading cycle.[^13^](#_ENREF_13) The calculated values of *a* and the measured values of *P*_c_ for each unloading/loading cycle are presented in Figure 3b. Substituting the obtained values of *a* and *P*_c_ into Equation (3) yielded the IFE (*G*_c_), which is a critical value of the applied strain energy release rate (*G*) and could be calculated as follows:[^12^](#_ENREF_12)

$G_{c}=\frac{12P_{c}^{2}a^{2}}{E'b^{2}h^{3}}\left(1+0.64\frac{h}{a}\right)^{2}.$ (3)

All tests were conducted in a laboratory air environment (40% RH) at 21°C.

1. Kim, H.-J. *et al.* High-durable agni nanomesh film for a transparent conducting electrode. *Small* **10**, 3767-3774 (2014).

2. Vosgueritchian, M. *et al.* Highly conductive and transparent pedot:Pss films with a fluorosurfactant for stretchable and flexible transparent electrodes. *Adv. Funct. Mater.* **22**, 421-428 (2012).

3. Nasibulin, A. G. *et al.* Multifunctional free-standing single-walled carbon nanotube films. *ACS Nano* **5**, 3214-3221 (2011).

4. Kim, U. J. *et al.* Graphene/carbon nanotube hybrid‐based transparent 2d optical array. *Adv. Mater.* **23**, 3809-3814 (2011).

5. De, S. *et al.* Silver nanowire networks as flexible, transparent, conducting films: Extremely high dc to optical conductivity ratios. *ACS Nano* **3**, 1767-1774 (2009).

6. Song, J.& Yu, J. Analysis of the t-peel strength in a cu/cr/polyimide system. *Acta materialia* **50**, 3985-3994 (2002).

7. Joo, S. C.& Baldwin, D. F. Analysis of adhesion and fracture energy of nano-particle silver in electronics packaging applications. *IEEE Trans. Adv. Packag.* **33**, 48-57 (2010).

8. Dauskardt, R. *et al.* Adhesion and debonding of multi-layer thin film structures. *Eng. Fract. Mech.* **61**, 141-162 (1998).

9. Lee, I. *et al.* Interfacial toughening of solution processed ag nanoparticle thin films by organic residuals. *Nanotechnology* **23**, 485704 (2012).

10. Kim, J. H. *et al.* Enhancing adhesion of screen‐printed silver nanopaste films. *Adv. Mater. Inter.* **2**, 1500283 (2015).

11. Yoon, T. *et al.* Direct measurement of adhesion energy of monolayer graphene as-grown on copper and its application to renewable transfer process. *Nano Lett.* **12**, 1448-1452 (2012).

12. Kanninen, M. An augmented double cantilever beam model for studying crack propagation and arrest. *Int. J. Fracture* **9**, 83-92 (1973).

13. Guyer, E. P.& Dauskardt, R. H. Electrical technique for monitoring crack growth in thin-film fracture mechanics specimens. *J. Mater. Res.* **19**, 3139-3144 (2004).
